# Modifying Robusta coffee aroma by green bean chemical pre-treatment

**DOI:** 10.1016/j.foodchem.2018.07.226

**Published:** 2019-01-30

**Authors:** Chujiao Liu, Qian Yang, Robert Linforth, Ian D. Fisk, Ni Yang

**Affiliations:** Division of Food Sciences, School of Biosciences, University of Nottingham, Sutton Bonington Campus, Loughborough, Leicestershire LE12 5RD, United Kingdom

**Keywords:** Coffee, Acetic acid, Pre-treatment, GC-MS, Sensory analysis, Aroma chemistry

## Abstract

Green Robusta beans were subjected to pre-treatment with the aim of reducing the perceived aroma difference between Arabica and Robusta coffee. Treatment was a short soaking procedure with varying concentrations of acetic acid (up to 5%). Samples were subjected to thermal treatment (roasted) and ground to a standardised particle size distribution. Aroma compounds were evaluated by headspace analysis using solid-phase microextraction and gas chromatography-mass spectrometry. Pre-treatment significantly affected aroma formation during roasting and resulted in a modified level of pyrazines, furanic compounds and sulfur-containing compounds (*p* < 0.05). Principal component analysis illustrated that the aroma profile of the pre-treated Robusta coffee was closer to the target Arabica coffee after roasting. Sensory results confirmed that the aroma of the 2% acetic acid pre-treated Robusta brew was similar to Arabica; the maximum inclusion level of Robusta coffee in a blend could be increased from 20% to 80%.

## Highlights

• Different levels of acetic acid were used to pre-treat Robusta green coffee beans.
• Acetic acid pre-treated Robusta had a more similar aroma profile to Arabica.
• The optimum level of acetic acid treatment was 2%.
• The maximum level of Robusta coffee added in a blend increased from 20% to 80%.

## 1 Introduction

According to the International Coffee Organization (2018a), global coffee consumption is 148 million cups per year and despite the background of global inflation, continued price fluctuations and restrictions on trade, there is a continued increase in demand for high quality coffee and speciality coffees (Bhumiratana, Adhikari, & Chambers Iv, 2011). The quality of coffee can be affected by many factors, these include growth conditions (Schnabel et al., 2017) and post-harvest treatment (Ameyu, 2017), in addition to preparation variables, such as roasting time-temperature profiles (Gloess et al., 2014, Yang et al., 2016) and water type (Clarke & Vitzthum, 2001).

Although more than 100 coffee species have been identified, *Coffea arabica* L. (Arabica coffee) and *Coffee canephora P*. (Robusta coffee) account for approximately 62% and 37% of all coffee produced globally (International Coffee Organization, 2018c). Arabica is considered to have a smooth, mild, and rich flavour, while Robusta possesses a flatter flavour, lacking in taste, with a muddy odour (Flament, 2002). However, Robusta coffee is less expensive (International Coffee Organization, 2018b), and is often blended with Arabica coffee beans to reduce cost, enhance crema formation (Folmer, Blank, & Hofmann, 2017) and create specific aroma profiles. However, the maximum level that can be included is often limited due to the loss of aroma quality (Dias et al., 2018, Frega et al., 2015). In the industry, it is common that coffee blends contain a mixture of 50/50 Arabica and Robusta (Dias et al., 2018). Therefore, there is a potential for up-valuing and improving the quality of Robusta coffee beans. This has the explicit goal of reducing the sensory difference, thereby making Robusta coffee flavour more like Arabica, increasing the range of choice for consumer at a lower final product cost.

The aroma of coffee is directly related to the chemical composition of the green coffee beans and typical coffee aromas are developed during the roasting process, due to complex reactions such as Maillard reactions, Strecker degradation, caramelisation, and oxidation (Fisk, Kettle, Hofmeister, Virdie, & Kenny, 2012). The Maillard reaction has been identified as one of the major pathways in the formation of volatile compounds during the coffee roasting process (Belitz and Grosch, 2009, Moon and Shibamoto, 2009, Van Boekel, 2006). During the Maillard reaction, reducing sugars, such as glucose and fructose, react with free amino acids to form N-substituted glycosylamine adducts (Schiff bases), which are then rearranged to aminoketones and aminoaldoses by Amadori and Heyns rearrangements (Poisson, Blank, Dunkel, & Hofmann, 2017). A complex reaction cascade of Amadori and Heyns rearrangement products leads to numerous volatile compounds and complex melanoidins (Poisson et al., 2017). Among numerous factors that can influence the generation of flavours by the Maillard reaction (Nursten, 2005), the pH at which the reaction is conducted influences the kinetics of certain reaction pathways, affecting the flavour of the final product (Jousse, Jongen, Agterof, Russell, & Braat, 2002). The influence of pH on the Maillard reaction and caramelisation in sugar-amino acid model systems has been previously studied. The earliest study observed the rate of d-glucose mutarotation can be accelerated by both hydroxyl ions and protons (Lowry, 1903). Wolfrom et al. reported that weak acids and conjugated bases are capable of promoting enolisation of the sugars, which may accelerate Mailard reaction and caramelization, as the enolised sugar is more easily dehydrated and fragmented than the ring form (Wolfrom, Cavalieri, & Cavalieri, 1947). Hodge (1953) later showed that sugar polymerisation can be strongly enhanced with increasing pH. In the model system, Amadori compounds are supposed to give rise preferentially to reductones and highly reactive dicarbonyls under alkaline conditions, while furfurals and related derivatives predominate under acidic conditions. Recent work by Nie et al. (2013) indicated that pH had a significant effect on thermally induced furan formation when the temperature is above 120 °C. Pre-treatment of green Robusta beans that modifies the internal pH could therefore alter flavour generation during the coffee roasting process (Penson et al., 2006).

In this study, green beans were pre-soaked in an aqueous acetic acid solution to affect aroma chemistry reaction pathways that occur during thermal treatment (roasting), with the overall aim of increasing the similarity of the aroma profiles of Arabica and Robusta coffee. Gas chromatography-mass spectrometry (GC-MS) with headspace solid-phase microextraction (SPME) was used to compare aroma compounds in treated roasted and ground (R & G) Robusta coffee, non-treated Robusta R & G and the target Arabica R & G coffee. Sensory analysis was used to determine the maximum percentage of Robusta that can be blended with Arabica without any perceived sensory difference.

## 2 Materials and methods

### 2.1 Coffee samples

Coffee beans were purchased from Edgehill Coffee, Warwick, United Kingdom. Robusta beans were single-origin washed beans from Vietnam, and Arabica beans were single-origin washed beans from Kenya. Twenty-five gram of green Robusta coffee beans were placed into a Modulyo Freeze Dryer 1311-03/08 JM (Edwards, Crawley, UK) at −40 °C for 72 h (to standardise the initial moisture content in green bean). Freeze-dried Robusta green beans were soaked in different concentrations of acetic acid (Sigma-Aldrich, Poole, UK): 0%, 1%, 2%, 3%, 4% or 5% for 2 h with three replicates each. The 0% acid-treated sample (treated with water only) was used as a control sample. Process/soaking loss is less than 0.5% dry weight of the green beans. Treated Robusta green beans, non-treated Robusta green beans and Arabica green beans were placed into a desiccator with saturated sodium nitrate solution (relative humidity 65.5%) at room temperature for 15 days to control the moisture content (around 11.5%). Determination of the water changes during pre-treatment and coffee roasting was carried out by weighing the coffee sample at every step.

Treated Robusta green beans, non-treated Robusta green beans and Arabica green beans (4 replicates each) were roasted at the same time in a convection oven (Mono Equipment, Swansea, UK). The roasting conditions used for green beans were 200 °C for 20 min (dark roast). Roasted samples were ground with an electronic coffee grinder (KG 49, Delonghi, Australia) then passed through a metal sieve size 710 μm (Endecotts, Essex, UK). All samples were sealed in non-permeable aluminium pouch (Protective Packaging Ltd, Cheshire, UK) and stored in the freezer at −80 °C until analysis.

### 2.2 pH measurement

Green/roasted coffee bean powder (2 g) was extracted with 15 mL of boiling water in a 30-mL tube, which was placed on a roller overnight (room temperature). The extracts were then filtered with No. 4 Whatman® filter paper before pH analysis. The pH of the extracts were measured using a pH meter (Inolab®).

### 2.3 Gas chromatography-mass spectrometry (GC-MS)

The ground coffee (1.5 g) was transferred into amber glass vials (20 mL) with four replicates for GC-MS analysis. An internal standard was prepared by adding 10 μL 3-heptanone (Sigma, Saint Louis, MO) into 10 mL methanol (Laboratory reagent grade; Fisher Scientific, Loughborough, UK). The internal standard (2 μL) was added to each coffee sample and kept for 1 h (25 ± 2 °C) prior to GC analysis. All analytical samples were randomised for GC-MS analysis.

A Trace 1300 series gas chromatograph coupled with a single-quadrupole mass spectrometer (Thermo Fisher Scientific, Hemel Hempstead, UK) was used for analysis of volatile aroma compounds. Samples were incubated at 40 °C for 5 min with shaking. A 50/30 μm DVB/CAR/PDMS SPME Fibre (Supelco, Sigma Aldrich, UK) was used to extract volatile aroma compounds from the sample headspace (extraction for 5 min then desorption for 2 min). The injector temperature was set at 200 °C in splitless mode (constant carrier pressure 18 psi).

Separation was carried out on a ZB-Wax capillary GC column (length 30 m, inner diameter 0.25 mm, film thickness 1 μm; Phenomenex Inc., Macclesfield, UK). Column temperature was held initially at 40 °C for 5 min, increased by 3 °C/min to 180 °C, then 8 °C/min to 240 °C and held for 2 min. Full scan mode was used to detect the volatile compounds (mass range from *m*/*z* 20 to 300) The method was modified from Yang et al. (2016) .

Volatile compounds were identified by comparison of their mass spectra and linear retention indices (LRI) of volatiles under the experimental conditions reported with literature data or with spectra in reference libraries (NIST/EPA/NIH Mass Spectral Library, version 2.0 g; National Institute of Standards and Technology, Gaithersburg, MD). The quantification of volatiles collected from the headspace was calculated from GC peak areas, by comparison with the peak area of the internal standard (3-heptanone). The equation used to measure the relative concentrations of the volatile compound in the coffee is shown below. The relative abundance of each volatile compound present in the headspace of Robusta or acetic acid-treated Robusta (average of 4) was calculated by dividing its peak area with its peak area in Arabica and then multiplying by 100.

$$\text{The}\;\text{relative}\;\text{concentration}\;\text{of}\;\text{volatile}\;\text{compounds}=\frac{\text{Peak}\;\text{area}\;\text{of}\;\text{volatile}\;\text{compound}\times \text{Amount}\;\text{of}\;\text{internal}\;\text{standard}\;(\text{mg})}{\text{Peak}\;\text{area}\;\text{of}\;\text{internal}\;\text{standard}\times \text{Dry}\;\text{weight}\;\text{of}\;\text{coffee}\;\text{powder}\;(\text{mg})}$$

### 2.4 Sensory evaluation

Two pre-treated Robusta coffee samples (2% and 4% acetic acid treated sample) were selected for sensory testing based on the analytical data. The coffee samples for sensory testing were freshly brewed in an 8-cup capacity cafetiere (French press) on the morning of the test day to avoid flavour loss. Following manufacturers’ instructions, 54 g of coffee were weighed and put in the cafetiere (Argos, Stafford, UK). Boiling water (860 mL) was then poured into the cafetiere, and stirred 5 times. The coffee was then left to brew for a strict 3 min. Coffee was then poured out of the cafetiere immediately into a glass beaker. Coffee brew (10 mL) was poured into amber vessels for each sample and served at ambient temperature (18 ± 2 °C). Amber glass vessels were used in this study to mask any colour differences.

All sensory tests were carried out in individual sensory booths under northern hemisphere lighting at the Sensory Science Centre of the University of Nottingham. 84 volunteers were recruited from student and staff at University of Nottingham to take part in this study. This study was approved by the School of Bioscience ethic committee at the University of Nottingham (ethics approval code: SBREC150118A).

For each triangle test, volunteers received three samples, and they were instructed to sniff the samples from left to right and identify which one was the different one (similar time interval for each sample) A 2-min break was given between triangle tests to allow nasal recovery. The presentation order between triangle test and within each triangle test was randomised. All the data were collected using Compusense Cloud (Compusense, Ontario, Canada).

Volunteers were invited for two testing sessions on two separate days within a week. A total of 15 triangle tests were conducted over two sessions; each session lasted approximately 45 min. The first session was to determine how different/similar Arabica coffee brew was compared to 2% acetic acid-treated Robusta, 4% acetic acid-treated Robusta and non-treated Robusta blended with Arabica. The second session was to determine how different/similar were the Arabica and Arabica blended with either 2% or 4% acid-treated Robusta (samples varied in blending ratio 20%, 40%, 60% and 80%).

### 2.5 Statistical analysis

Relative abundance of each aroma compound was analysed by ANOVA (according to the Jarque-Bera Normality test, all aroma and pH data were normally distributed) to identify if a significant difference (*p* < 0.05) existed for each compound between Arabica samples, non-treated Robusta samples and treated Robusta samples. All results were analysed by IBM® SPSS® Statistics version 21.0.0 and Microsoft Excel 2010, using samples as the fixed effect, and a Tukey’s HSD post-hoc test. Principal component analysis (PCA) was performed by Excel XLStat Version 2015.5.01.23373.

Compusense Cloud was used in the sensory study for data collection and analysis. Number of responses were compared to the critical table in ISO (2007) to determine significance; α = 0.05 was selected for difference testing, α = 0.2, β = 0.05, p_D_ = 30% were selected for similarity testing.

## 3 Results and discussion

### 3.1 Determination of the aroma compounds in Robusta beans with different pre-treatment methods

Twenty-four aroma compounds were identified within all the coffee samples (Table 1). Volatile aroma compounds included 2 furanic compounds, 2 organic acids, 4 heterocyclic compounds (N-containing), 3 sulfur-containing compounds, 1 aldehyde, 1 ketone and 12 pyrazines. Linear retention index, identification method and related attributes found for the aroma compounds were determined (Table 1). The detailed information for 24 aroma compounds is illustrated in the Supplementary material (CAS number, molecular weight, significant fragments, probability from NIST database, odour threshold and chromatogram obtained by SPME-GC-MS analysis). The compounds shown in bold (Supplementary material) are potent odorants and all compounds have been previously reported in coffee (Boothroyd et al., 2014, Clarke and Vitzthum, 2001, Flament, 2002, Illy and Viani, 2005, Mottram, 2018, Poisson et al., 2017). The relative abundance of each volatile compound presented in the headspace of the eight coffee samples is shown in Table 2, which all indicated significant differences (*p* < 0.05) between treated and non-treated samples (Table 2).

**Table 1:** Linear retention index, identification method and odour description for 24 aroma compounds.

	aroma compound	LRI	Literature LRI^a^	Identification method	odour description^b^	functional group
1	hexanal	1106	1024–1087	MS, STD	grassy, green, oily	aldehyde
2	trimethylthiazole	1100	1000	MS	cocoa, roasted-like	sulfur-containing
3	3-methylthiophene	1144	1117	MS	ash	sulfur-containing
4	1-methylpyrrole	1168	1123	MS	green, beany, smokey,-tarry	heterocyclic N
5	1-ethylpyrrole	1211	–	MS	burnt	heterocyclic N
6	pyrazine	1226	1192–1214	MS, STD, L	pungent, sweet, floral	pyrazine
7	methylpyrazine	1261	1260–1288	MS, STD	nutty, roasted, sweet, chocolatey	pyrazine
8	4-methylthiazole	1298	1282	MS	tomato, fruity, nutty, green	sulfur-containing
9	hydroxyacetone	1305	1294–1308	MS	pungent, sweet-caramellic, burnt	ketone
10	2,5-dimethylpyrazine	1355	1316	MS, STD, L	nutty, roasted, grassy, corn	pyrazine
11	2,6-dimethylpyrazine	1361	1319	MS, STD, L	nutty, sweet, fried	pyrazine
12	ethylpyrazine	1368	1323–1325	MS, STD	nutty, roasted	pyrazine
13	2,3-dimethylpyrazine	1381	1335	MS, STD, L	nutty, roasted, green	pyrazine
14	2-ethyl-6-methylpyrazine	1418	1363–1381	MS, STD, L	roasted, hazelnut-like	pyrazine
15	2-ethyl-5-methylpyrazine	1426	1383	MS, STD, L	roasted, hazelnut-like	pyrazine
16	trimethylpyrazine	1437	1387–1412	MS	nutty, roasted	pyrazine
17	2,6-diethylpyrazine	1470	–	MS, L	hazelnut-like	pyrazine
18	3-ethyl-2,5-dimethylpyrazine	1480	1466–1469	MS	hazelnut-like, earthy, baked, roasty	pyrazine
19	acetic acid	1484	1435–1459	MS, STD, L	sour	organic acid
20	furfural	1504	1447–1466	MS, STD, L	pungent, sweet, caramellic, bread-like	furans
21	pyrrole	1553	1487–1504	MS	nutty, hay-like, herbaceous	heterocyclic N
22	propanoic acid	1569	1487–1574	MS	sour, cheese, butter	organic acid
23	1-methyl-2-formylpyrrole	1664	1610–1626	MS	burnt	heterocyclic N
24	2-furanmethanol	1668	1573–1682	MS, STD, L	caramellic, burnt, smoky	furans

^a&b^Literature LRI and odour description are taken from Boothroyd et al., 2014, Flament, 2002, Mottram, 2018.

LRI = linear retention index; Identification method (MS = mass spectrum compared to NIST database, or L = literature; STD = standard compound).

CAS number, molecular weight, significant fragments, probability from NIST database, odour threshold and chromatogram obtained by SPME-GC-MS analysis for the aroma compounds are available in Supplementary Material.

**Table 2:** Average relative abundance of each volatile compound (relative to Arabica) presented in the headspace was calculated in the eight coffee samples (excludes acetic acid).

	aroma compound	Robusta	0%	1%	2%	3%	4%	5%	Arabica
1	2,6-diethylpyrazine	498 ± 7.9^e^	338 ± 35^d^	278 ± 24^cd^	246 ± 23^bcd^	235 ± 20^bc^	174 ± 35^ab^	208 ± 22^bc^	100 ± 17^a^
2	3-ethyl-2,5-dimethylpyrazine	435 ± 39^d^	340 ± 49^cd^	266 ± 22^bc^	256 ± 20^bc^	221 ± 8.6^b^	176 ± 19^ab^	226 ± 18^b^	100 ± 18^a^
3	2-ethyl-6-methylpyrazine	327 ± 21^d^	276 ± 27^cd^	233 ± 14^bc^	210 ± 18^bc^	197 ± 16^bc^	148 ± 25^ab^	183 ± 38^ab^	100 ± 26^a^
4	trimethylpyrazine	314 ± 27^d^	244 ± 23^cd^	193 ± 26^bc^	176 ± 17^abc^	160 ± 19^ab^	123 ± 12^ab^	150 ± 29^ab^	100 ± 14^a^
5	2-ethyl-5-methylpyrazine	302 ± 22^d^	265 ± 17^cd^	228 ± 24^bcd^	207 ± 22^bc^	197 ± 17^bc^	153 ± 21^ab^	187 ± 36^abc^	100 ± 20^a^
6	ethylpyrazine	230 ± 21^c^	185 ± 16^bc^	172 ± 34^abc^	150 ± 26^ab^	144 ± 19^ab^	118 ± 19^ab^	141 ± 27^ab^	100 ± 16^a^
7	2,5-dimethylpyrazine	223 ± 13^b^	165 ± 5.8^b^	149 ± 23^ab^	123 ± 16^a^	122 ± 12^a^	86 ± 11^a^	104 ± 12^a^	100 ± 13^a^
8	2,6-dimethylpyrazine	204 ± 21^c^	181 ± 17^bc^	154 ± 27^abc^	134 ± 26^ab^	130 ± 25^ab^	98 ± 23^a^	112 ± 18^ab^	100 ± 11^a^
9	methylpyrazine	169 ± 16^b^	128 ± 15^ab^	111 ± 4.6^a^	91 ± 5.9^a^	90 ± 9.8^a^	76 ± 17^a^	79 ± 10^a^	100 ± 14^a^
10	2,3-dimethylpyrazine	178 ± 23^c^	137 ± 12^bc^	113 ± 6.2^ab^	104 ± 9.2^ab^	93 ± 6.5^ab^	77 ± 8.3^a^	92 ± 8.6^ab^	100 ± 8.2^ab^
11	pyrazine	124 ± 3.1^d^	102 ± 5.1^c^	76 ± 2.6^b^	68 ± 9.0^ab^	62 ± 8.6^ab^	60 ± 8.3^ab^	54 ± 6.8^a^	100 ± 3.4^c^
12	furfural	94 ± 8.2^a^	108 ± 4.2^ab^	149 ± 20^abc^	152 ± 23^abc^	201 ± 22^c^	154 ± 19^abc^	174 ± 12^bc^	100 ± 4.1^a^
13	2-furanmethanol	34 ± 12^ab^	29 ± 3.9^a^	39 ± 6.9^ab^	48 ± 7.1^ab^	55 ± 6.9^ab^	58 ± 6.3^ab^	65 ± 12^b^	100 ± 5.6^c^
14	4-methylthiazole	192 ± 2.3^c^	133 ± 17^b^	89 ± 2.2^a^	77 ± 11^a^	68 ± 15^a^	63 ± 16^a^	64 ± 12^a^	100 ± 3.3^ab^
15	trimethylthiazole	107 ± 5.1^c^	88 ± 6.2^ab^	84 ± 1.9^ab^	82 ± 9.3^ab^	77 ± 2.0^a^	73 ± 2.1^a^	73 ± 3.5^a^	100 ± 2.3^bc^
16	3-methylthiophene	96 ± 11^bc^	82 ± 17^abc^	78 ± 4.1^ab^	71 ± 1.2^a^	64 ± 3.1^a^	73 ± 2.1^a^	62 ± 2.3^a^	100 ± 3.1^c^
17	propanoic acid	37 ± 6.2^a^	45 ± 8.1^ab^	59 ± 2.3^bc^	66 ± 5.3^c^	69 ± 4.7^c^	66 ± 5.1^c^	68 ± 1.1^c^	100 ± 5.9^d^
18	hydroxyacetone	107 ± 5.9^ab^	132 ± 14^b^	103 ± 6.1^ab^	97 ± 5.2^ab^	108 ± 12^ab^	89 ± 2.4^a^	84 ± 5.1^a^	100 ± 8.7^ab^
19	hexanal	710 ± 23^c^	667 ± 36^bc^	876 ± 20^d^	475 ± 52^b^	607 ± 47^bc^	494 ± 5.2^b^	520 ± 55^bc^	100 ± 15^a^
20	1-methyl-2-formylpyrrole	264 ± 6.9^b^	131 ± 17^a^	128 ± 5.0^a^	99 ± 2.8^a^	102 ± 1.7^a^	93 ± 8.1^a^	80 ± 12^a^	100 ± 14^a^
21	pyrrole	247 ± 15^e^	160 ± 7.2^d^	106 ± 3.5^c^	96 ± 10^abc^	84 ± 5.2^ab^	81 ± 7.9^ab^	78 ± 3.2^a^	100 ± 4.2^bc^
22	1-methylpyrrole	100 ± 1.6^c^	55 ± 2.2^b^	46 ± 5.6^ab^	35 ± 7.0^ab^	34 ± 1.1^a^	33 ± 7.5^a^	29 ± 2.6^a^	100 ± 2.4^c^
23	1-ethylpyrrole	94 ± 5.9^b^	52 ± 7.9^a^	55 ± 8.7^a^	44 ± 3.3^a^	45 ± 4.2^a^	42 ± 3.5^a^	39 ± 9.1^a^	100 ± 3.2^b^

^abcde^Samples with the same letter code in any row are not significantly different (*p* < 0.05).

Mean ± standard deviation.

### 3.2 Evaluation of pH and acetic acid in Robusta, treated Robusta and Arabica

Coffee brew acidity is an important sensory quality attribute (Clarke & Vitzthum, 2001) and the pH of the Robusta green bean and roasted coffee was significantly higher than Arabica. The pH of the coffee bean extract increased with roasting (Table 3). The pH of green bean was significantly lower than the pH of the roasted bean. The increase in pH during roasting may be explained by the degradation of organic acids in green beans and formation of compounds during the initial stage of roasting (such as chlorogenic acids and citric acid). This is consistent with the results of Gloess et al. (2014). There was no significant difference between the untreated Robusta and the process control (0%), however pH significantly reduced with increasing concentrations of acetic acid treatment (*p* < 0.05). As expected, the pH of acetic acid-treated green beans reduced significantly compared with non-treated Robusta. There was no significant difference between green Arabica and treated green Robusta when 2–5% acid treatment levels were used. There was no significant difference between roasted Arabica and roasted Robusta when 4–5% acid treatment levels were used.

**Table 3:** pH and relative abundance of acetic acid in Arabica, Robusta and acetic acid-treated Robusta coffee (0, 1, 2, 3, 4, and 5% acetic acid).

	green bean extract pH	roasted bean extract pH	relative abundance for acetic acid
Arabica	4.42 ± 0.09^ab^	5.05 ± 0.03^a^	100 ± 12.6^b^
Robusta	4.86 ± 0.05^d^	5.55 ± 0.01^d^	52 ± 8.4^a^
0%	4.84 ± 0.04^d^	5.51 ± 0.01^d^	65 ± 4.4^a^
1%	4.6 ± 0.02^c^	5.34 ± 0.02^c^	102 ± 16.1^b^
2%	4.47 ± 0.06^b^	5.23 ± 0.08^bc^	170 ± 39.1^b^
3%	4.45 ± 0.03^b^	5.2 ± 0.03^b^	316 ± 19.5^c^
4%	4.42 ± 0.02^ab^	5.13 ± 0.04^ab^	326 ± 11.3^c^
5%	4.39 ± 0.01^a^	5.01 ± 0.03^a^	391 ± 6.7^d^

^abcd^Samples with the same letter code in any column are not significantly different (*p* < 0.05).

Mean ± standard deviation.

The relative abundances of acetic acid in non-treated Robusta, treated Robusta and Arabica sample are shown in Table 3. Non-treated Robusta (acetic acid 52 ± 8.4) and 0% acetic acid treated Robusta (acetic acid 65 ± 4.4) had significantly lower concentrations of acetic acid when compared with Arabica (acetic acid 100 ± 12.6) (*p* < 0.05). There was no significant difference in relative abundance of acetic acid for both 1% and 2% acetic acid-treated Robusta when compared with Arabica sample. However, at higher treatment levels (3–5%) the treated coffee had significantly higher levels of acetic acid than Arabica.

### 3.3 Summary of all coffee samples via principal component analysis

**Fig. 1:**
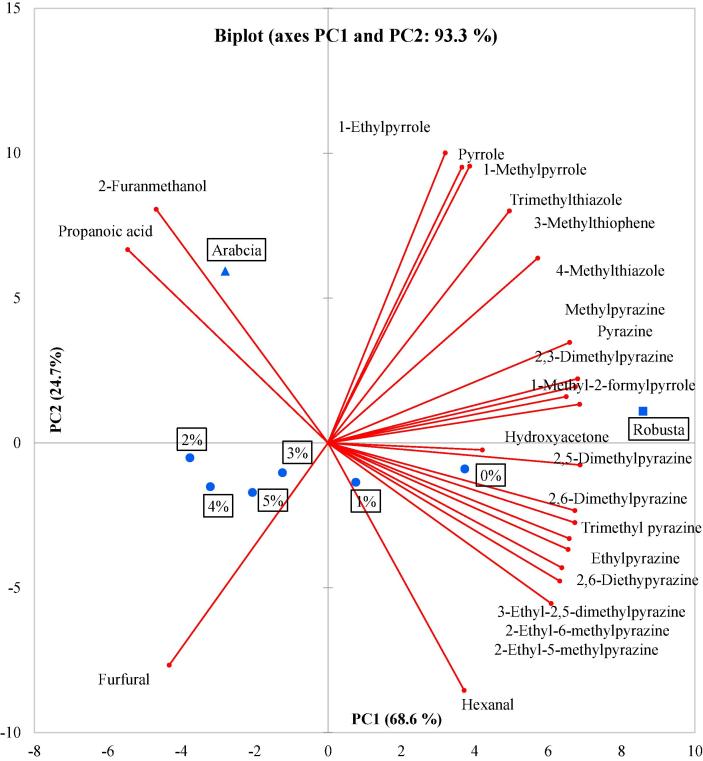
Principle component analysis (Bi-plot) of the aroma of Arabica, Robusta and acetic acid treated Robusta coffee (0, 1, 2, 3, 4, and 5% acetic acid).

Principal component analysis (PCA) was used to illustrate the differences between the 23 aroma compounds (excluding acetic acid) across the 8 R & G samples (Fig. 1). The first principal component (PC1) accounted for 68.6% of the variance in the dataset and showed a trend with increasing number of pyrazines from left to right, Arabica (left) was clearly separated from Robusta (right). The second principal component (PC2) accounted for 24.7% of the variance and showed separation between the treated Robusta and untreated Robusta samples. Pyrroles, pyrazines and hydroxyacetone were positively correlated with PC1, and furfural and propanoic acid were negatively correlated with PC1. There were visible differences between Arabica and Robusta, characterised by these samples being located in different corners of the PCA plot. The non-treated Robusta sample had greater levels of pyrazines when compared with the other samples. There was no discrimination between 2% acetic acid-treated Robusta and 4% acetic acid-treated Robusta which were located together in the centre of the plot.

### 3.4 Pyrazine content in acetic acid-treated Robusta samples

Pyrazines belong to a class of heterocyclic nitrogen-containing compounds, which contain four carbon and two nitrogen atoms in a ring skeleton and are one of the most important roast aromas in a large number of thermally treated foods (Nursten, 2005). According to Silwar and Lullmann (1993), pyrazine formation in Arabica after roasting is lower than Robusta; this is due to the content of free amino acids being lower in green Arabica than in Robusta. Since pyrazines account for about 14% of the total volatile compounds in roasted coffee (Flament, 2002), it is important in our study to understand pyrazine formation. A reduction in pyrazine concentration could make the aroma profile of Robusta more similar to that of Arabica.

**Fig. 2:**
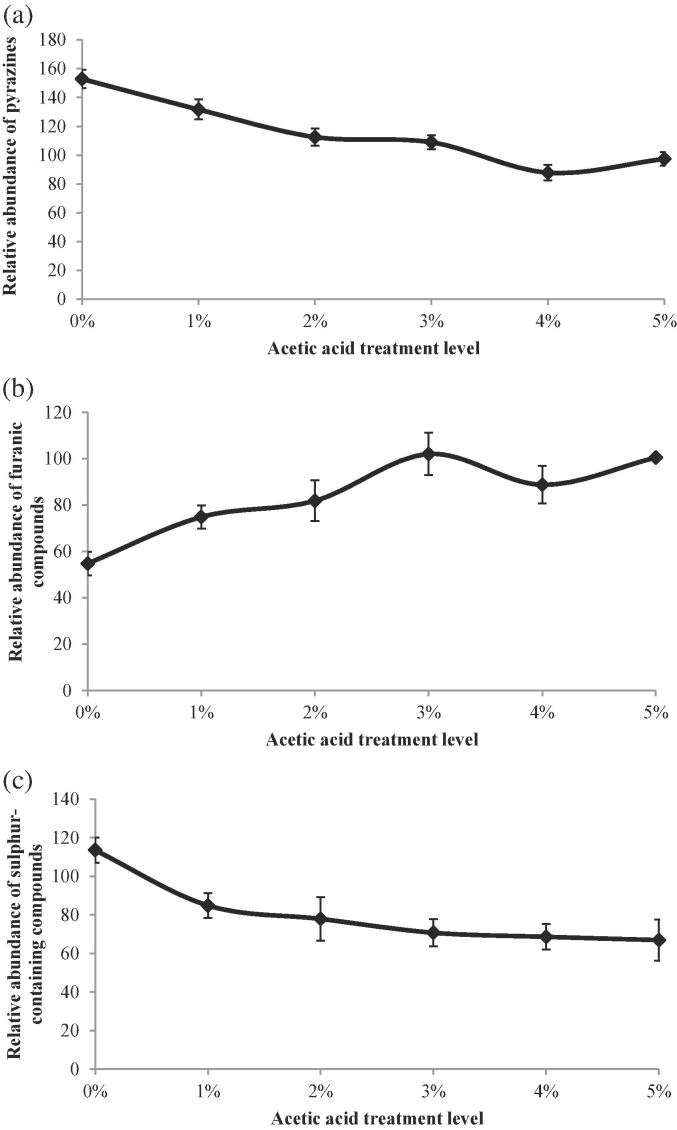
(a) Relative abundance of pyrazines (relative to Arabica) for acetic acid treated Robusta (non-treated Robusta was 193.2 ± 1.8 (mean ± standard deviation)); (b) Relative abundance of furanic compounds (relative to Arabica) for acetic acid treated Robusta (non-treated Robusta was 53.2 ± 5.2); (c) Relative abundance of sulphur-containing compounds (relative to Arabica) for acetic acid treated Robusta (non-treated Robusta sample was 142.9 ± 6.2). Error bars represent for standard deviation.

A group of pyrazines was identified and their relative abundances are shown in Table 2. The relative abundances of all selected pyrazines were higher in Robusta than Arabica but significantly reduced by acid pre-treatment (Table 2) (*p* < 0.05). For the four pyrazines, 2,5-dimethylpyrazine, 2,6-dimethylpyrazine, ethylpyrazine and 2,3-dimethylpyrazine, there was no significant difference between the Arabica control and the 1% acetic acid-treated Robusta. The relative abundance of total pyrazines reduced significantly (*p* < 0.05) as the levels of acetic acid in the soaking solution increased (Fig. 2(a)). There were no significant differences between the Arabica and the 2%, 3%, 4% and 5% acetic acid-treated Robusta samples (*p* ≥ 0.05).

The first step of pyrazine generation is regarded as condensation of the carbonyl and amine groups to yield a Schiff base (Shahidi, Ho, & Van Chuyen, 2013). The Schiff base rearranges to the Amadori product. The initial step formation of the Schiff base is one of the rate-limiting steps (Nursten, 2005). The reaction rate of the amine is highly dependent upon the pH of the sample; the maximum rate of most amine reactions occurs in an alkaline environment (Westheimer, 1939). This is due to the nitrogen becoming more nucleophilic at higher pH. Pre-soaking the green Robusta coffee beans with acetic acid decreased the green bean pH (from 4.86 ± 0.05 to 4.39 ± 0.01). At lower pH conditions the rate of carbonyl-amine condensation reactions would be decreased significantly because of the relatively low reactivity of the protonated amine group (Wong & Bernhard, 1988). Nursten also reported the consequence of protonation and deprotonation of various moieties of the carbohydrates (Nursten, 2005). After loss of water from the amine and carbonyl groups, fragmentation of the carbohydrate occurs through reverse aldol, enolisation, and dehydration reactions (Shibamoto & Bernhard, 1977). According to the literature, reverse aldol, enolisation and dehydration are all base catalysed (Mosher, 1992, Shibamoto and Bernhard, 1977). These results are consistent with acidic conditions limiting the production of pyrazines by reducing the reactivity of the amino group (Koehler & Odell, 1970).

### 3.5 Furanic compounds content in acetic acid-treated Robusta samples

Furfural and 2-furanmethanol were measured in all samples from R & G coffee (Table 2). There was a significantly lower relative amount of furanic compounds in non-treated Robusta samples when compared with Arabica (*p* < 0.05). The relative abundance of furfural and 2-furanmethanol increased by about 2-fold in the 5% acetic acid-treated Robusta when compared with the non-treated Robusta. The total relative abundance of furanic compounds significantly increased (*p* < 0.05) as the concentration of acetic acid in the soaking solution increased (Fig. 2(b)). There were no significant differences between Arabica and 3%, 4%, 5% acetic acid-treated Robusta samples (*p* ≥ 0.05). These results indicate that acidic conditions favoured the formation of furfural and 2-furanmethanol. In a model system involving l-ascorbic acid and l-threonine/l-serine the total furans formation increased with increasing acidity (Yu & Zhang, 2010). Previous studies also indicated that the concentrations of furans and furfural increased significantly as the pH was reduced (Madruga & Mottram, 1998).

Furan products are markers of sugar degradation (Nursten, 2005). According to Flament (2002), furfural is formed by thermal degradation of glucose and was shown to be present in cysteine/glucose model systems. However, formation could also be from Amadori pentose compounds and an intermediate 3-deoxyosone. This compound provides sweet, caramel like but also cinnamon-almond like aroma. 2-Furanmethanol was also found in heated cysteine/glucose model systems producing a warm caramel like odour.

Generally, roasted Arabica beans have higher levels of furfural and 2-furanmethanol when compared with roasted Robusta beans. This is due to Arabica having a higher sucrose content. The increase in furfural and 2-furanmethanol as a result of the pre-treatment method therefore reduces the difference between Arabica and Robusta beans.

### 3.6 Sulfur-containing compounds content in acetic acid-treated Robusta samples

Sulfur-containing compounds were identified in the R & G samples and the relative abundance of 3-methylthiophene, trimethylthiazole and 4-methylthiazole are presented in Table 2. All sulfur-containing compounds were significantly reduced (*p* < 0.05) in the treated samples (Table 2). There were no significant differences between acetic acid-treated Robusta and Arabica in the levels of 4-methylthiazole. The relative abundance of 4-methylthiazole was significantly higher in non-treated Robusta when compared with Arabica. Non-treated Robusta sample showed no significant difference when compared with the Arabica control in trimethylthiazole and 3-methylthiophene (*p* ≥ 0.05). The total relative abundance of sulfur-containing compounds showed a decreasing trend as the concentration of acetic acid in the soaking solution increased (Fig. 2(c)). However, process control sample (0%) also indicated a significantly reduced (*p* < 0.05) level of sulfur-containing compounds compared with the Robusta control sample.

The formation of sulfur-containing volatile compounds is due to Maillard reactions and Strecker degradation involving sulfur-containing amino acids (Nursten, 2005, Schenker et al., 2002). The main amino acids involved in the formation of sulfur-containing volatile compounds are cysteine and methionine (Flament, 2002, McGorrin, 2011). Since most of these amino acids are water-soluble, they could be lost during the green beans pre-treatment process.

### 3.7 Sensory evaluation

**Fig. 3:**
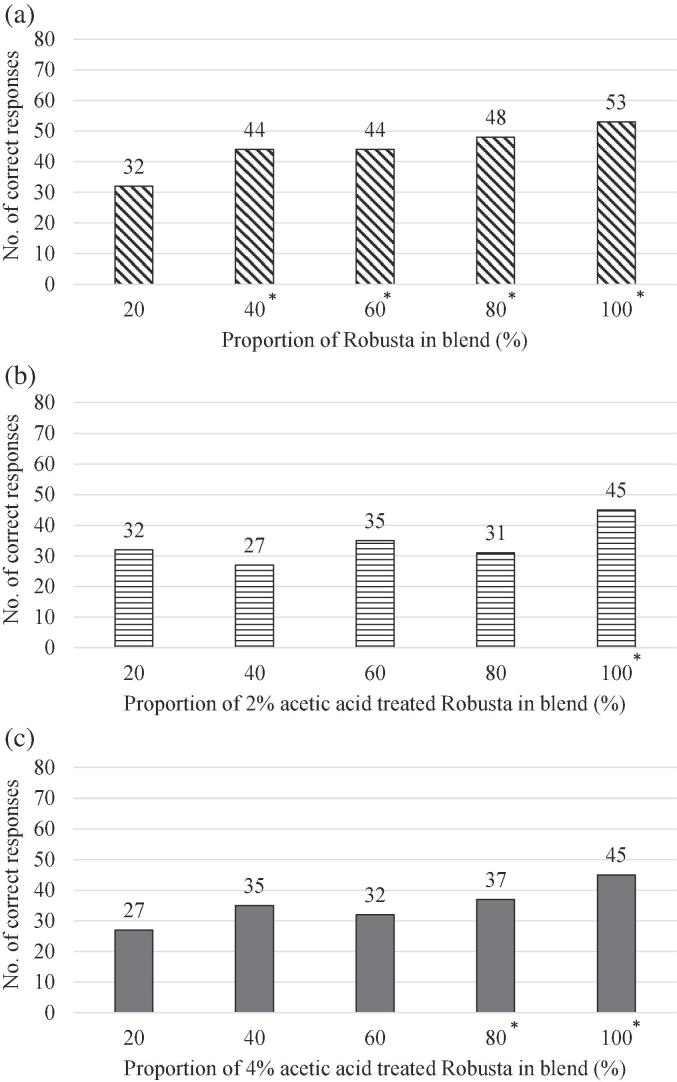
Sensory testing to identify similarity of 20%, 40%, 60% and 80% Robusta blends with the Arabica control (a) non-treated Robusta (b) 2% acetic acid treated Robusta (c) 4% acetic acid treated Robusta. Proportion of discrimination = 30%, β = 0.05; blended samples are similar to Arabica if the number of correct response is less than 36 out of 84 responses, * indicates a significant difference compare with the Arabica control.

The aim of the sensory study was to determine the maximum percentage of Robusta that can be blended with Arabica without any perceived sensory difference. Robusta coffee, and 2% and 4% acetic acid-treated Robusta coffee, were blended with up to 80% Arabica and compared with the Arabica control, the results for the numbers of correct responses are shown in Fig. 3. Numbers of correct responses were compared to critical tables in ISO (2007); samples were classed as being similar to Arabica if the number of correct responses was less than 36 out of 84.

Participants could not discriminate the aroma of Arabica and 20% Robusta blended with Arabica (number of correct responses was 32; Fig. 3(a)). However, as the blending ratio was increased to 40% and above, participants started to constantly perceive a difference. When 2% acetic acid-treated Robusta was used, participants could not discriminate any difference up to 80% Robusta inclusion when compared with Arabica (Fig. 3(b)). At 4% acetic acid treatment, 60% blending could be achieved with no significant difference in aroma (Fig. 3(c)). However, when it increased to 80% and above, participants began to perceive a difference.

## 4 Conclusions

In conclusion, this study has developed a pre-treatment method for Robusta coffee with an improved aroma profile that is closer to the aroma of Arabica. Principal component analysis showed the acetic acid-treated Robusta coffee had a closer aroma profile to Arabica. Sensory testing showed that the maximum proportion of Robusta that could be added to Arabica increased from 20% for the non-treated coffee to 60% for the 4% acetic acid-treated and 80% for the 2% acetic acid-treated coffee. The treatment of Robusta with acetic acid before roasting clearly alters its aroma chemistry and allows it to be blended with Arabica in greater proportions.

The proposed process is a fundamental study designed to demonstrate the principle of modifying green bean chemistry to control aroma formation after roasting. Commercial implementation will first need to include taste evaluations and consider the need to adhere to the coffee extracts and chicory extracts (England) regulations (2000).
