# Supplementary material for: Modifying Robusta coffee aroma by green bean chemical pre-treatment
Source: Food Chem. 2019 Jan 30;272:251–7. doi: 10.1016/j.foodchem.2018.07.226 (PMC6191532; doi:10.1016/j.foodchem.2018.07.226)
Supplement: Supplementary Data 1 [file mmc1.docx]

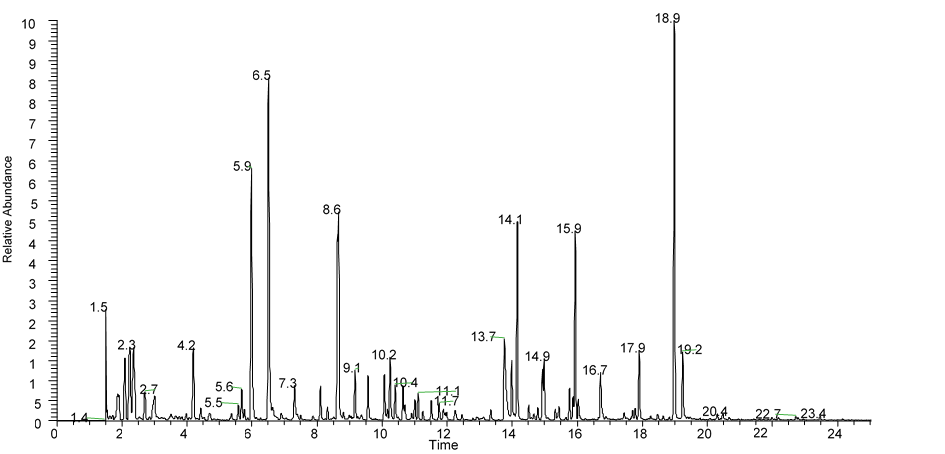


(a)


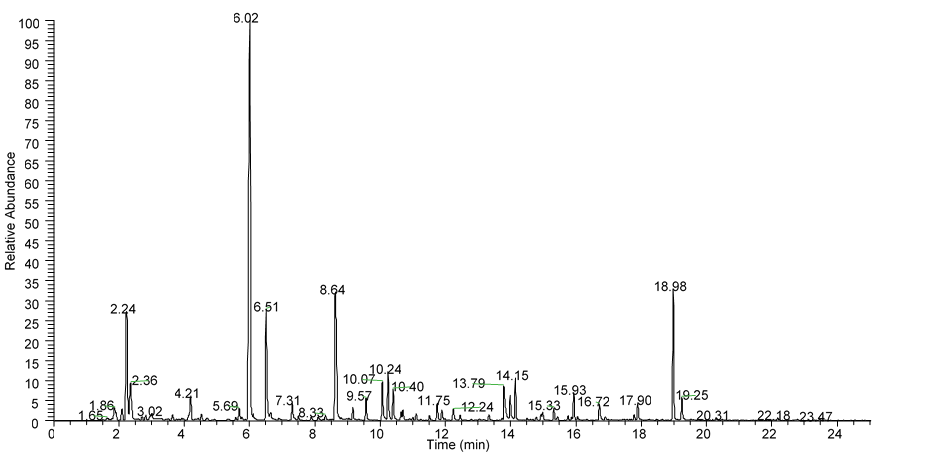


(b)


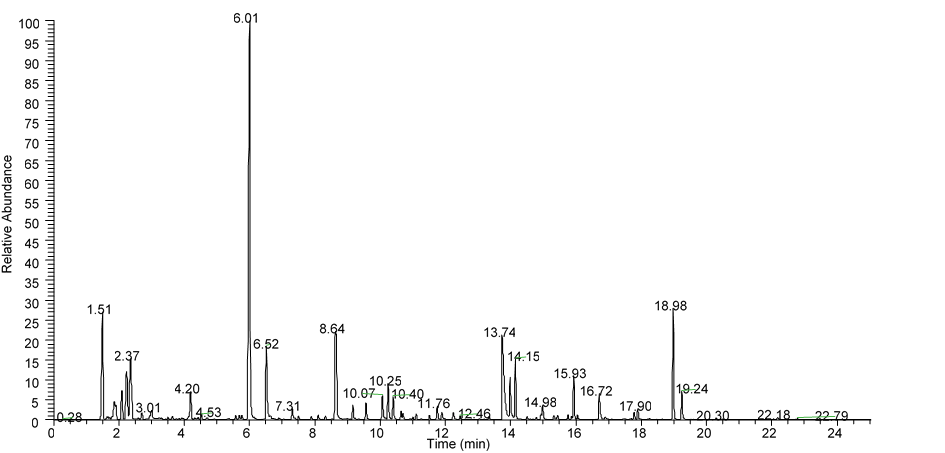


(c)

Figure S1 Chromatogram obtained by SPME-GC-MS analysis of volatile compounds in (a) Arabica; (b) non-treated Robusta; (c) 2% treated Robusta coffee. Numbers indicate the retention times (min).

Table S1 CAS number, molecular weight, significant fragments, identification method, probability from NIST database and odour threshold (Mottram, 2018) for 24 aroma compounds in roasted coffee samples.

|  | **Aroma Compound** | **CAS** | **Mol. WT** (g/mol) | **Significant fragments** | **Probability from MS** (%) | **Odour threshold** (ppb) |
| --- | --- | --- | --- | --- | --- | --- |
| 1 | **Hexanal** | 000066-25-1 | 100.16 | 44, 41, 56, 57 | 86 | 17 |
| 2 | **Trimethylthiazole** | 013623-11-5 | 127.21 | 59, 71, 86, 112, 127 | 72 | 50 |
| 3 | **3-Methylthiophene** | 000616-44-4 | 98.16 | 45, 97, 98, 99 | 71 | 5000-8500 |
| 4 | 1-Methylpyrrole | 000096-54-8 | 81.12 | ,53, 78, 80, 81, | 89 | - |
| 5 | 1-Ethylpyrrole | 000617-92-5 | 95.15 | 39, 67, 80, 95 | 51 | - |
| 6 | **Pyrazine** | 000290-37-9 | 80.09 | 53, 80 | 56 | 175000 |
| 7 | **Methylpyrazine** | 001333-41-1 | 93.13 | 40, 53, 67, 94 | 92 | 60000 |
| 8 | **4-Methylthiazole** | 000693-95-8 | 99.15 | 39, 45, 71, 72, 99 | 89 | 300 |
| 9 | **Hydroxyacetone** | 007339-87-9 | 136.15 | 31, 43, 74 | 78 | 100000 |
| 10 | **2,5-Dimethylpyrazine** | 000123-32-0 | 108.14 | 39, 40, 42, 81, 108 | 80 | 800-1700 |
| 11 | **2,6-Dimethylpyrazine** | 000108-50-9 | 108.14 | 38, 40, 42, 108 | 92 | 54000 |
| 12 | **Ethylpyrazine** | 013925-00-3 | 108.14 | 39, 53, 80, 107 | 87 | 6000 |
| 13 | **2,3-Dimethylpyrazine** | 005910-89-4 | 108.14 | 40, 67, 108 | 83 | 2500 |
| 14 | **2-Ethyl-6-Methylpyrazine** | 013925-03-6 | 122.17 | 39, 56, 94, 121 | 75 | 20 |
| 15 | **2-Ethyl-5-methylpyrazine** | 013360-64-0 | 122.17 | 39, 56, 94, 122 | 72 | 100 |
| 16 | **Trimethylpyrazine** | 014667-55-1 | 122.17 | 39, 42, 81, 122 | 70 | 9000 |
| 17 | 2,6-Diethylpyrazine | 013067-27-1 | 136.19 | 39, 53, 108, 120, 135 | 71 | 6 |
| 18 | **3-Ethyl-2,5-dimethylpyrazine** | 015707-34-3 | 136.19 | 39, 42, 56, 108, 135 | 53 | 43000 |
| 19 | **Acetic acid** | 000064-19-7 | 60.05 | 43, 45, 60 | 97 | 2000 |
| 20 | **Furfural** | 000098-01-1 | 96.09 | 39, 96 | 91 | 3000-23000 |
| 21 | **Pyrrole** | 000109-97-7 | 67.09 | 39, 40, 41, 67 | 87.9 | 49600 |
| 22 | Propanoic acid | 000079-09-4 | 74.08 | 45, 57, 73, 74 | 96.4 | 20000 |
| 23 | **1-Methyl-2-formylpyrrole** | 001192-58-1 | 109.13 | 53, 80, 109 | 45.9 | 37 |
| 24 | **2-Furanmethanol** | 000098-00-0 | 98.1 | 41, 53, 81, 97, 98 | 87 | 5000 |
